# Special footwear designed for pregnant women and its effect on kinematic gait parameters during pregnancy and postpartum period

**DOI:** 10.1371/journal.pone.0232901

**Published:** 2020-05-12

**Authors:** Marta Gimunová, Martin Zvonař, Martin Sebera, Pavel Turčínek, Kateřina Kolářová

**Affiliations:** 1 Department of Kinesiology, Faculty of Sports Studies, Masaryk University, Brno, Czech Republic; 2 Department of Informatics, Faculty of Business and Economics, Mendel University, Brno, Czech Republic; West Virginia University, UNITED STATES

## Abstract

During pregnancy, an array of changes occurs in women body to enable the growth and development of the future baby and the consequent delivery. These changes are reflected in the range of motion of trunk, pelvis, lower limbs and other body segments, affect the locomotion and some of these changes may persist to the postpartum period. The aim of this study was to describe the changes affecting the gait during pregnancy and to determine the effect of tested footwear on kinematic gait characteristics during pregnancy as previous studies indicate that special orthopaedic insoles and footwear might be useful in prevention of the common musculoskeletal pain and discomfort related to pregnancy. Participants from the control group (n = 18), without any intervention, and the experimental group (n = 23), which was wearing the tested shoes, were measured at their 14, 28 and 37 gestational weeks and 28 weeks postpartum to capture the complete pregnancy-related changes in gait. The gait 3D kinematic data were obtained using Simi Motion System. The differences between the control and experimental group at the first data collection session in most of the analysed variables, as well as relatively high standard deviations of analysed variables indicate large individual differences in the gait pattern. The effect of tested footwear on kinematic gait pattern changes may be explained by its preventive effect against the foot arches falling. In the control group, changes associated previously with the foot arches falling and hindfoot hyperpronation were observed during advanced phases of pregnancy and postpartum, e.g. increase in knee flexion or increase in spinal curvature. For the comprehensive evaluation of the tested footwear on pregnancy gait pattern, future studies combining the kinematic and dynamic plantographic methods are needed.

## Introduction

Non-fitting shoes have a negative effect on muscles and bones and may lead to foot pain [1]. During pregnancy, increase in foot swelling, and volume is related to an increase in foot length and width. Furthermore, decrease in foot arches height, and changes in plantar pressure distribution during the gait during pregnancy were observed in previous studies [2–6]. Due to increased volume of the foot and foot arches height decrease, choosing suitable shoes may be difficult. A number of previous studies and patents focused on special footwear designed for pregnant women and its effect on the foot discomfort, and pain reduction is discussed among both, researchers and shoemakers [7–10]. However, the number of scientific studies investigating the effect of special insoles or footwear on the gait during pregnancy longitudinally and on a large sample is lacking.

During pregnancy, a decrease in a single-support time, stride length and an increase in double-support time and step width have been reported previously [11–13]. Furthermore, a decreased range of motion of the trunk, anterior displacement of the center of mass, posterior inclination of thoracic segment, anterior pelvic tilt, increased lumbar lordosis, head posteriorization, knee hyperextension, lowering of the medial longitudinal plantar arch, increased volume, length and width of the foot, greater medio-lateral sway and greater lateral rotation of the feet have been observed in the gait in late pregnancy [5, 14]. However, the results of the previous studies are sometimes contradictory, suggesting large inter-individual differences in pregnancy adaptations. The contradictory findings may also be a result of different methodological approaches used in previous studies [15].

In previous studies, the effect of special shoes and silicone insoles on pregnancy walking and feet was investigated [7, 8]. When wearing balanced inclined shoes, a decrease in plantar pressure moments and an increase bloodstream velocity was observed, suggesting that this type of footwear may decrease the excessive load on the feet and improve the foot blood circulation [7]. In a study by Marques, Goncalves, Santos and Vilas-Boas [8] on the comfort and functionality of pregnant women’s feet, hindfoot and complete silicone insoles were tested. Wearing hindfoot insole did not significantly changed the mean pressure values of the hindfoot but increased them in the forefoot. The complete insole efficiently redistributed the pressure values and decreased the maximum pressure, which increased the comfort of the feet of pregnant women [8].

As mentioned above, previous studies of special footwear designed for pregnant women focused mostly on plantar pressure distribution changes. However, those changes significantly affect also the gait kinematics as reported in the previous study by Gimunová et al. [16]. The purpose of this study was to assess the effect of tested footwear comparing the kinematic gait parameters changes during the pregnancy and postpartum period in an experimental group which was wearing tested shoes and control group of pregnant women which was measured without any intervention.

## Materials and methods

50 women participated in the study; however, because of pre-term births, tiredness and swelling in the last trimester of pregnancy, relocation into another city and other personal reasons, 23 and 18 women completed all four measurement sessions for the experimental and control group, respectively. The inclusion criteria consisted of a suitable week of pregnancy (<14 g.w.), singleton pregnancy and age between 18 to 40 years. Exclusion criteria consisted of physical limitation affecting the movement and twin or triplet pregnancies. The participants were recruited at their first gestation weeks by advertisement of this study at local gynaecologists and by a social media advertisement. Informed consent was obtained from all participants before participating in the study. The Ethical Board of the Faculty of Sports Studies, Masaryk University, Brno, Czech Republic approved the study.

All women participated in the measurements three times during the pregnancy (at 14 ± 3.1, 28 ± 2.6 and 37 ± 1.25 gestation week) and once after the delivery (28 ± 9.9 weeks postpartum). The criterium for the postpartum measurement was reaching back the pre-pregnancy body mass (± 3 kg). The experimental group received two pairs of special footwear and insoles two weeks after the first measurement and was instructed to wear them at least 3 hours per day. Dividing participants into the experimental and control group was done randomly.

**Fig 1:**
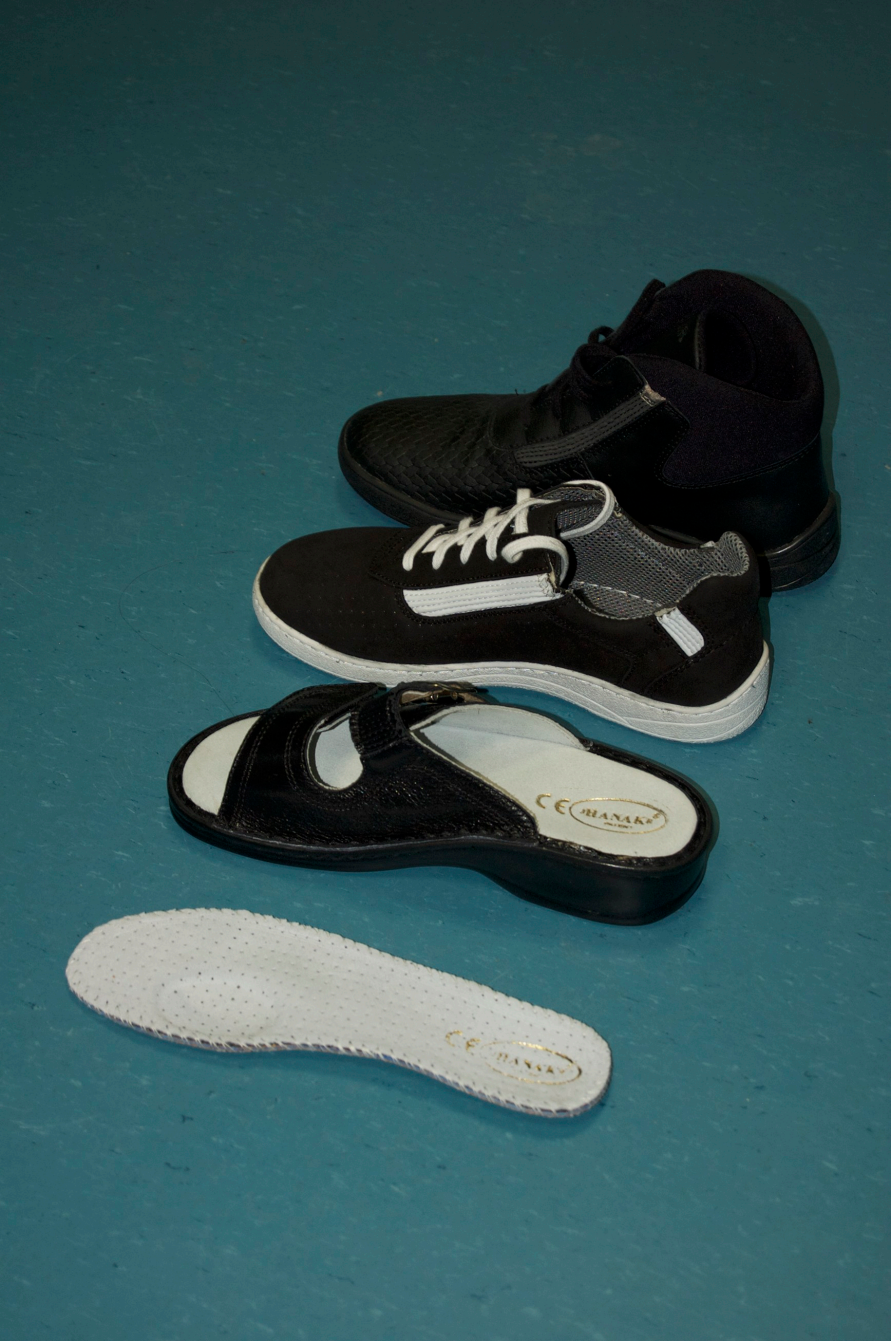
Experimental insole and footwear.

Tested footwear and insoles J Hanák R (Snovídky, Czech Republic) are designed to help with redistribution of forces acting on foot. The insoles, US Patent US 20150013189 A1, most prominent features are a depression under the first metatarsophalangeal joint, elastic leather straps, which are sewn into the shoe upper sole at the instep and the heel, and a depression under the heel [17–19]. The tested footwear and insoles (Fig 1) were obtained from the producer *Boty J Hanák R*, *s*.*r*.*o*., shoe type Active, high top Active and sandal shoes type 304.

In the following table, mean age (years), height (m) and body mass (kg) ± SD at the 14, 28 and 37 weeks of gestation and 28 weeks postpartum of both the experimental (n = 23) and control (n = 18) group are shown (Table 1).

**Table 1:** Participants’ characteristics.

	Age	Height	Body mass 14g.w.	Body mass 28 g.w.	Body mass 37 g.w.	Body mass postpartum
**Control group**	32.25±3.43	169.75±4.81	65.65±9.99	73.65±10.74	78.44±11.07	64.88±10.42
**Experimental group**	28.94±3.22	167.72±6.48	65.28±7.79	71.77±8.04	76.13±9.02	65.75±7.24

The participants were asked to walk barefoot along a 6-meter walkway with a full-body marker set, wearing their underwear or a fitting top and shorts five times at each data collection session at their self-selected speed; however, only one gait cycle (the second) from one trial (the third trial if no unexpected change of direction happened during this trial) was used for further analysis. This gait cycle was chosen as during the third trial participants became used to the walking path with cameras and therefore performed their natural gait pattern.

The gait 3D kinematic data were obtained using 8 cameras (Basler A602fc) from the Simi Motion System (Unterschleißheim, Germany), filmed at 100 Hz. Full body marker set using 8 lightweight retroreflective markers with a diameter of 10 mm was used in this study. Retroreflective markers were placed on anthropometric points left and right acromiale, iliospinale anterius, tibiale laterale and malleolus lateralis.

**Fig 2:**
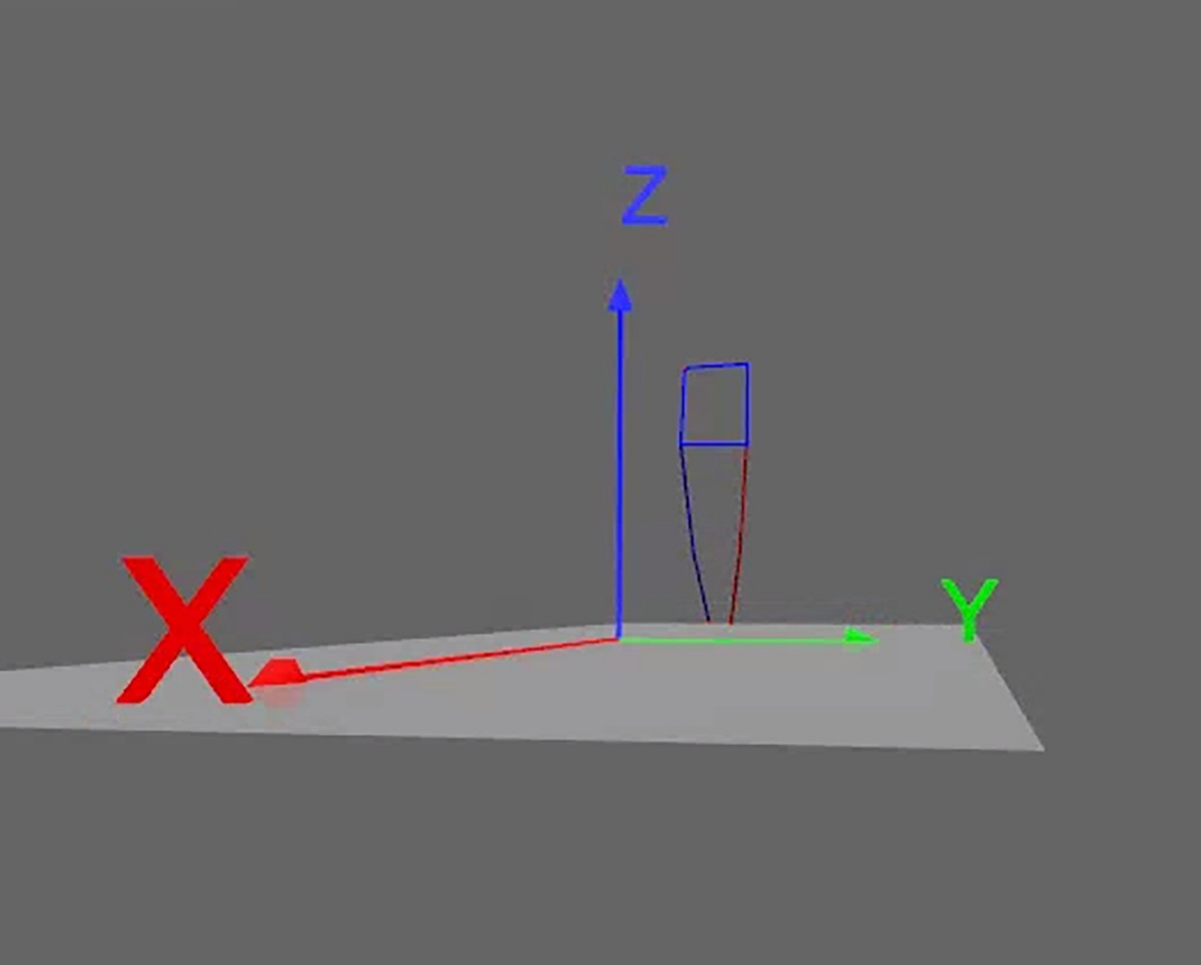
3D model stick diagram.

Tracking, a semi-automatic markers digitalization of one step of the right limb and one step of the left limb was done prior to the 3D model creation (Fig 2) using the Simi Motion Software, version 7.5.292. The beginning of the step cycle was determined by the heel strike (the first foot contact of the with ground).

The following variables were exported from the 3D models: (i) anterio-posterior trunk angle (the angle between marker acromiale, iliospinale anterius and saggital plane), (ii) spinal curvature change (manifested by the height of marker acromile), (iii) hip joint angle (the angle defined by marker acromiale, iliospinale anterius and tibiale laterale in saggital plane), and (iv) knee joint angle (the angle defined by marker iliospinale anterius, tibiale laterale and malleolus lateralis in saggital plane).

This study was based on dissertation thesis research [20].

### Statistical analysis

Most of the variables did not meet the assumptions of normal distribution and homogeneity of variance, verified by tests of normality and Levene test of homogeneity of variances. Therefore, to compare the differences between different gestational weeks of pregnancy and postpartum period for the control and experimental group, Friedman test and Wilcoxon matched pairs test were used. P < 0.05 was considered to be statistically significant. To compare differences between the control and experimental group at the 14, 28 and 37 weeks of gestation (g.w.) and 28 weeks postpartum effect size obtained by Cohen’s d was used. Cohen's d is interpreted as ≥ 0.20 small, ≥ 0.50 medium or clinically significant, and ≥ 0.80 large effect [21]. The statistics were obtained using the Statistica Statsoft 12 and Microsoft Excel.

## Results

### Participants’ characteristics

The effect size comparison between the experimental and control group showed large effect (Cohen’s d = 1.00) in age, small effect (Cohen’s d = 0.35) in body height, no effect in body mass at 14 g.w. and 28 weeks postpartum (Cohen’s d = 0.04 and 0.10, respectively) and a small effect in body mass at 28 g.w. and 37 g.w.(Cohen’s d = 0.20 and 0.23, respectively).

### Step cycle time

Means and standard deviations of step cycle time at 14, 28 and 37 g.w. and 28 weeks postpartum for the experimental and control group and their effect size comparison are shown in Table 2.

**Table 2:** Step cycle time (s) means and standard deviations of the experimental and control group at 14, 28 and 37 g.w. and 28 weeks postpartum and their effect size comparison.

	Experimental group		Control group		Experimental-Control group Cohens’ d (CI)
	Mean	SD	Mean	SD	
**14 g.w.**	1.07	0.09	1.06	0.08	0.12 (0.08; 0.15)
**28 g.w.**	1.08	0.07	1.08	0.05	0.00 (-0.03; 0.02)
**37 g.w.**	1.10	0.09	1.08	0.07	**0.25 (0.21; 0.28)**
**postpartum**	1.04	0.07	1.07	0.06	**-0.46 (-0.49; -0.43)**

With advancing pregnancy, a small increase in step cycle time was observed in both groups. However, statistically significant differences were found only in the experimental group comparing 28 and 37 g.w. with 28 weeks postpartum (p = 0.006 and p = 0.004, respectively). The results of Cohen’s d show no significant differences between the groups at the 14 and 28 g.w. However, a small effect was found comparing the experimental and control group at the 37 g.w. and 28 weeks postpartum.

### Kinematic variables

Tables 3 and 4 shows means, maximums and minimums and standard deviations of analysed kinematic variables. Tables 5 and 6 shows the results of statistical analysis between different data collection sessions of the experimental and control group for the left and right side, respectively. Table 7 shows the effect size comparison between the experimental and control group.

**Table 3:** Mean, maximum, minimum and standard deviation of the left side for the experimental and control group at 14, 28 and 37 g.w. and 28 weeks postpartum.

			14 g.w.	28 g.w.	37 g.w.	Postpartum
**Anterio-posterior trunk angle (°)**	**experimental group**	**mean**	-0.36 ± 9.86	-0.32 ± 10.12	2.36 ± 8.44	2.75 ± 12.04
		**max**	3.62 ± 9.68	2.42 ± 8.21	4.67 ± 7.27	5.05 ± 12.56
		**min**	-4.37 ± 10.45	-3.60 ± 13.22	0.13 ± 9.21	0.10 ± 10.70
	**control group**	**mean**	-9.08 ± 10.12	-7.56 ± 7.82	-8.36 ± 8.33	-8.12 ± 14.85
		**max**	-5.67 ± 9.64	-4.31 ± 7.21	-5.36 ± 7.83	-3.61 ± 15.87
		**min**	-11.94 ± 10.79	-10.65 ± 8.58	-11.06 ± 8.88	-13.96 ± 10.40
**Spinal curvature (m)**	**experimental group**	**mean**	1.34 ± 0.12	1.31 ± 0.16	1.33 ± 0.10	1.38 ± 0.07
		**max**	1.36 ± 0.12	1.34 ± 0.14	1.35 ± 0.10	1.41 ± 0.08
		**min**	1.32 ± 0.12	1.27 ± 0.19	1.31 ± 0.10	1.35 ± 0.06
	**control group**	**mean**	1.41 ± 0.17	1.34 ± 0.19	1.40 ± 0.05	1.36 ± 0.06
		**max**	1.46 ± 0.24	1.38 ± 0.17	1.42 ± 0.05	1.39 ± 0.07
		**min**	1.36 ± 0.11	1.30 ± 0.21	1.36 ± 0.05	1.32 ± 0.06
**Hip joint angle (°)**	**experimental group**	**mean**	160.65 ± 9.48	161.70 ± 11.82	161.90 ± 8.64	155.62 ± 22.66
		**max**	173.09 ± 7.58	174.79 ± 10.07	175.59 ± 5.69	170.23 ± 14.95
		**min**	145.19 ± 12.43	143.92 ± 13.66	144.97 ± 10.01	137.44 ± 28.66
	**control group**	**mean**	162.87 ± 3.55	164.84 ± 2.72	162.94 ± 4.38	158.66 ± 11.49
		**max**	175.44 ± 3.14	176.89 ± 2.25	175.55 ± 3.57	172.38 ± 10.22
		**min**	145.66 ± 5.03	147.58 ± 4.51	145.77 ± 6.13	137.77 ± 19.90
**Knee joint angle (°)**	**experimental group**	**mean**	160.52 ± 4.96	159.01 ± 7.99	160.32 ± 6.01	157.28 ± 19.43
		**max**	176.09 ± 3.58	175.46 ± 5.56	176.97 ± 3.11	172.01 ± 18.61
		**min**	125.80 ± 7.73	126.29 ± 9.73	126.40 ± 6.62	126.19 ± 16.47
	**control group**	**mean**	162.47 ± 1.73	162.46 ± 3.09	163.36 ± 4.05	159.63 ± 8.44
		**max**	175.35 ± 1.87	175.20 ± 2.34	176.00 ± 2.26	174.53 ± 2.74
		**min**	130.30 ± 5.63	131.36 ± 9.59	133.77 ± 11.39	125.85 ± 15.98

**Table 4:** Mean, maximum, minimum and standard deviation of the right side for the experimental and control group at 14, 28 and 37 g.w. and 28 weeks postpartum.

			14 g.w.	28 g.w.	37 g.w.	Postpartum
**Anterio-posterior trunk angle (°)**	**experimental group**	**mean**	-2.32 ± 6.93	-1.70 ± 5.33	-3.59 ± 4.63	-4.27 ± 5.59
		**max**	1.16 ± 6.96	0.51 ± 4.85	-1.57 ± 4.17	1.41 ± 17.91
		**min**	-6.63 ± 8.89	-4.26 ± 6.35	-5.81 ± 5.63	-7.84 ± 6.70
	**control group**	**mean**	-10.42 ± 9.46	-9.35 ± 6.03	-9.44 ± 6.54	-9.58 ± 13.83
		**max**	-7.54 ± 9.03	-6.58 ± 5.76	-6.55 ± 5.95	-4.06 ± 15.78
		**min**	-13.38 ± 10.11	-12.59 ± 6.76	-12.83 ± 7.94	-16.17 ± 10.45
**Spinal curvature (m)**	**experimental group**	**mean**	1.34 ± 0.12	1.30 ± 0.16	1.33 ± 0.10	1.34 ± 0.18
		**max**	1.36 ± 0.12	1.33 ± 0.14	1.35 ± 0.10	1.38 ± 0.11
		**min**	1.31 ± 0.12	1.27 ± 0.19	1.30 ± 0.10	1.29 ± 0.27
	**control group**	**mean**	1.42 ± 0.17	1.35 ± 0.19	1.40 ± 0.05	1.35 ± 0.07
		**max**	1.46 ± 0.23	1.39 ± 0.17	1.43 ± 0.05	1.38 ± 0.07
		**min**	1.37 ± 0.11	1.30 ± 0.21	1.37 ± 0.05	1.30 ± 0.07
**Hip joint angle (°)**	**experimental group**	**mean**	163.24 ± 3.87	159.78 ± 13.37	162.24 ± 8.72	156.77 ± 18.76
		**max**	176.17 ± 3.07	173.85 ± 9.32	174.61 ± 8.84	171.20 ± 12.41
		**min**	144.79 ± 9.83	140.85 ± 21.69	146.44 ± 9.93	138.71 ± 22.59
	**control group**	**mean**	163.04 ± 3.38	165.36 ± 2.52	164.66 ± 4.23	159.77 ± 13.88
		**max**	175.99 ± 2.95	177.52 ± 1.46	177.46 ± 2.58	173.40 ± 11.60
		**min**	145.72 ± 4.00	147.66 ± 4.89	147.90 ± 4.81	142.65 ± 18.56
**Knee joint angle (°)**	**experimental group**	**mean**	159.66 ± 5.54	157.68 ± 12.13	158.18 ± 9.82	157.48 ± 17.75
		**max**	175.47 ± 4.22	174.12 ± 7.57	174.90 ± 5.53	171.04 ± 16.76
		**min**	125.34 ± 9.02	124.03 ± 12.47	125.23 ± 13.73	127.00 ± 18.76
	**control group**	**mean**	162.10 ± 2.43	160.94 ± 3.44	159.70 ± 4.63	157.61 ± 19.96
		**max**	175.31 ± 2.56	175.34 ± 2.31	175.55 ± 2.68	170.55 ± 17.44
		**min**	129.49 ± 5.21	128.56 ± 5.42	125.49 ± 8.80	125.65 ± 22.56

**Table 5:** Statistical analysis results of the kinematic variables, left side.

		Experimental group			Control group		
		Mean	Max	Min	Mean	Max	Min
		p value	p value	p value	p value	p value	p value
**Anterio-posterior trunk angle**	**14–28 g.w.**	0.637	0.987	0.338	0.982	0.844	0.878
	**14–37 g.w.**	0.927	0.808	0.484	0.913	0.878	0.647
	**14 g.w.—postpartum**	0.411	0.784	0.113	0.913	0.556	0.445
	**28–37 g.w.**	0.831	0.584	0.584	0.420	0.349	0.616
	**28. g.w.—postpartum**	0.465	0.447	0.260	0.248	0.214	0.231
	**37 g.w.—postpartum**	0.670	0.761	0.605	0.420	0.878	0.285
**Spinal curvature**	**14–28 g.w.**	0.638	0.808	0.638	0.215	0.184	0.327
	**14–37 g.w.**	0.927	1.000	0.784	0.472	0.446	0.286
	**14 g.w.—postpartum**	0.042*	0.011*	0.153	0.112	0.145	0.199
	**28–37 g.w.**	0.927	0.808	0.879	0.372	0.420	0.112
	**28. g.w.—postpartum**	0.018*	0.012*	0.024*	0.085	0.170	0.122
	**37 g.w.—postpartum**	0.121	0.023*	0.191	0.048*	0.085	0.025*
**Hip joint angle**	**14–28 g.w.**	0.592	0.168	0.485	0.064	0.133	0.157
	**14–37 g.w.**	0.648	0.162	0.738	0.500	0.744	0.616
	**14 g.w.—postpartum**	0.976	0.738	0.465	0.085	0.349	0.085
	**28–37 g.w.**	0.855	0.976	0.485	0.094	0.133	0.102
	**28. g.w.—postpartum**	0.429	0.107	0.738	0.003*	0.022*	0.014*
	**37 g.w.—postpartum**	0.523	0.094	0.429	0.420	0.500	0.102
**Knee joint angle**	**14–28 g.w.**	0.548	0.277	0.961	0.557	0.679	0.500
	**14–37 g.w.**	0.378	0.078	0.903	0.711	0.306	0.616
	**14 g.w.—postpartum**	0.951	0.224	0.412	0.039*	0.122	0.248
	**28–37 g.w.**	0.378	0.212	0.951	0.286	0.133	0.170
	**28. g.w.—postpartum**	0.627	0.236	0.378	0.170	0.053	0.267
	**37 g.w.—postpartum**	1.000	0.026*	0.248	0.094	0.043*	0.102

**Table 6:** Statistical analysis results of the kinematic variables, right side.

		Experimental group			Control group		
		Mean	Max	Min	Mean	Max	Min
		p value	p value	p value	p value	p value	p value
**Anterio-posterior trunk angle**	**14–28 g.w.**	0.808	0.570	0.661	0.845	0.557	0.845
	**14–37 g.w.**	0.627	0.191	0.927	0.647	0.711	0.711
	**14 g.w.—postpartum**	0.212	0.101	0.316	0.744	0.913	0.396
	**28–37 g.w.**	0.162	0.094	0.260	0.616	0.647	0.845
	**28. g.w.—postpartum**	0.029*	0.089	0.089	0.058	0.711	0.048*
	**37 g.w.—postpartum**	0.136	0.191	0.068	0.184	0.879	0.122
**Spinal curvature**	**14–28 g.w.**	0.638	0.783	0.485	0.396	0.267	0.845
	**14–37 g.w.**	0.808	0.761	0.976	0.199	0.231	0.094
	**14 g.w.—postpartum**	0.128	0.068	0.236	0.085	0.157	0.039*
	**28–37 g.w.**	0.584	0.715	0.523	0.396	0.879	0.102
	**28. g.w.—postpartum**	0.064	0.033*	0.114	0.039*	0.112	0.039*
	**37 g.w.—postpartum**	0.330	0.212	0.394	0.002*	0.008*	0.002*
**Hip joint angle**	**14–28 g.w.**	0.685	0.445	0.783	0.018	0.102	0.170
	**14–37 g.w.**	0.784	0.808	0.330	0.102	0.053	0.145
	**14 g.w.—postpartum**	0.121	0.029*	0.584	0.811	0.845	0.586
	**28–37 g.w.**	0.738	0.605	0.447	0.744	0.811	0.845
	**28. g.w.—postpartum**	0.605	0.523	0.670	0.001*	0.018*	0.306
	**37 g.w.—postpartum**	0.083	0.068	0.101	0.078	0.145	0.267
**Knee joint angle**	**14–28 g.w.**	0.961	0.236	0.961	0.267	0.983	0.879
	**14–37 g.w.**	0.879	0.784	0.362	0.007*	0.711	0.020*
	**14 g.w.—postpartum**	0.715	0.006*	0.073	0.913	0.231	0.711
	**28–37 g.w.**	0.627	0.287	0.153	0.249	0.528	0.157
	**28. g.w.—postpartum**	0.503	0.202	0.024*	0.058	0.170	0.286
	**37 g.w.—postpartum**	0.447	0.055	0.412	0.102	0.043*	0.170

**Table 7:** Results of Cohen’s d comparison of the means of kinematic variables between the experimental and control group at 14, 28, 37 g.w. and 28 weeks postpartum.

	14 g.w.	28 g.w.	37 g.w	Postpartum
	Cohen's d (CI)	Cohen's d (CI)	Cohen's d (CI)	Cohen's d (CI)
**Anterio-posterior trunk angle**	**1.01 (-1.82; 5.38)**	**1.36 (-0.82; 4.14)**	**1.07 (-0.82; 4.09)**	**0.58 (-1.71; 6.97)**
**Lateral trunk motion**	**-0.35 (-2.43; 1.12)**	**-0.36 (-3.81; 1.24)**	**-0.41 (-3.74; 0.94)**	**-0.33 (-10.01; 1.01)**
**Spinal curvature**	**-0.56 (-0.61; -0.49)**	**-0.29 (-0.35; -0.20)**	**-0.90 (-0.94; -0.87)**	-0.08 (-0.15; -0.04)
**Hip joint angle**	0.05 (-1.53; 1.62)	**-0.65 (-6.11; 0.52)**	**-0.36 (-3.92; 1.60)**	-0.18 (-7.85; 6.23)
**Knee joint angle**	**-0.58 (-2.85; 0.54)**	**-0.39 (-5.35; 1.20)**	**-0.20 (-4.21; 1.94)**	-0.01 (-7.26; 9.21)

### Anterio-posterior trunk

Statistical analysis showed significant difference between 28 g.w. and postpartum measurement in both groups for the right side. The anterio-posterior trunk angle mean lowest value occurred at the 28 g.w. Results of Cohen’s d showed significant differences between the experimental and control group at all data collection sessions.

### Spinal curvature change

In the experimental group, statistically significant differences were observed between the 14 g.w. and postpartum in mean and maximum (p = 0.042 and p = 0.011, respectively), between 28 g.w. and postpartum in mean, maximum and minimum (p = 0.018, p = 0.012 and p = 0.024, respectively) and between 37 g.w. and postpartum in maximum (p = 0.023) for the left side. For the right side, statistically significant difference was observed between 28 g.w. and postpartum in maximum (p = 0.033).

In the control group, statistically significant differences were observed between the 37 g.w. and postpartum in mean and minimum (p = 0.048, p = 0.025, respectively) for the left side. For the right side, statistically significant differences were observed between 14 g.w. and postpartum in minimum (p = 0.039), 28 g.w. and postpartum in mean and minimum (p = 0.039 for both) and between 37 g.w. and postpartum in mean, maximum and minimum (p = 0.002, p = 0.008 and p = 0.002, respectively).

Results of Cohen’s d showed significant differences between the experimental and control group at all pregnancy data collection sessions, which may be explained by different mean heights of the groups.

### Hip joint angle

In the experimental group, statistically significant difference was found between 14 g.w. and postpartum in minimum (p = 0.029) for the right side. In the control group, statistically significant differences were found between 28 g.w. and postpartum in mean, maximum and minimum (p = 0.003, p = 0.022 and p = 0.014, respectively) for the left side. For the right side, in the control group, statistically significant differences were found between 14 and 28 g.w. in mean (p = 0.018) and between 28 g.w. and postpartum in mean and maximum (p = 0.001 and p = 0.018, respectively).

Results of Cohen’s d showed no significant differences between the experimental and control group at 14 g.w. and postpartum. During the second and third trimester of pregnancy significant differences between the experimental and control group were observed.

### Knee joint angle

In the experimental group, statistically significant difference was observed between 37 g.w. and postpartum in maximum (p = 0.026) for the left side. For the right side, statistically significant differences were observed between 14 g.w. and postpartum in maximum (p = 0.006) and between 28 g.w. and postpartum in minimum (p = 0.024). For the experimental group, at the postpartum compared to pregnancy period a decrease in maximal knee extension was observed. In the control group, statistically significant differences were observed between 14 g.w. and postpartum in mean (p = 0.039) and between 37 g.w. and postpartum in maximum (p = 0.043) for the left side. For the right knee, statistically significant differences were observed between 14 and 37 g.w. in mean and minimum (p = 0.007 and p = 0.020, respectively) and between 37 g.w. and postpartum in maximum (p = 0.043). As in the experimental group, at the postpartum compared to pregnancy period a decrease in maximal knee extension was observed in the control group. Additionally, increase in maximal knee flexion was observed at 37 g.w. in the control group.

Results of Cohen’s d showed significant differences between the experimental and control group at 14, 28 and 37 g.w. During postpartum period, no significant differences between the experimental and control group were observed.

## Discussion

The aim of this study was to determine the effect of tested footwear on kinematic gait characteristics during pregnancy and postpartum period.

### Body mass

The mean weight gain during the followed pregnancy period was 12.79 kg and 10.85 for the control and experimental group, respectively. Observed weight gains meet the current recommendation for healthy women who have a normal weight (BMI 18.5–24.9) consisting of 11.5–16 kg during the pregnancy, as the mean weight gain during the first trimester usually consists of < 2 kg [22]. After four to six months postpartum, women return to their pre-pregnancy weight [23]. In this study, the difference between 14 g.w. and 28 weeks postpartum consisted of less than 1 kg. Similarly, in population-based studies, an average weight gain between pregnancies consisting of 1–2 kg has been observed [24, 25].

### Step cycle time

At advanced stages of pregnancy, an increase in step cycle time suggesting a slower gait velocity was observed in both groups; however, statistically significant difference was found only in the experimental group comparing advanced stages of pregnancy and postpartum period. A slower velocity at advanced stages of pregnancy was observed in previous studies as well [11, 26–28]. Previous studies suggested that walking at slower velocity may be preferred to maximize safety during the gait [27].

In the control group, no statistically significant differences in step cycle time during pregnancy and postpartum period were found. Similarly, no significant difference in gait velocity between late pregnancy and one year postpartum was observed also in a study by Foti et al. [29].

### Trunk

For the anterio-posterior trunk angle, statistically significant differences were found between the 28 g.w. and postpartum in both groups, suggesting a restricted anterio-posterior trunk range of motion at 28 g.w. Statistically significant reduction in trunk maximum forward flexion was also observed in a previous study [30]; however, no change in sagittal plane kinematics of the trunk was also observed previously [12]. The decrease in the trunk range of motion is usually explained by increased abdominal volume. Similarly to this study, in which significant differences between the experimental and control group were observed at all data collection sessions, a large variance in sagittal plane kinematics of the trunk between subjects, indicating an individual dynamic response to the increased inertial effects of pregnancy, was observed previously [12].

In the current study, spinal curvature change was represented by the change in acromiale height. In the experimental group, data suggest a statistically significant straightening of the spinal curvature, i.e. increase in acromiale height at the postpartum period compared to pregnancy. In the control group data show a significant increase in spinal curvature, i.e. decrease in acromiale height during postpartum period compared to pregnancy.

As mentioned above, a large variance in individual posture adaptations was observed during pregnancy. Both increase and decrease in lumbar and thoracic curvatures have been reported previously [29, 31]. Furthermore, increased thoracic curvature has been associated with an increase in shoulders internal rotation, which may also contribute to the observed decrease in acromiale height. Besides, no change or a decrease in lumbar lordosis, reported previously during pregnancy, may result in a slight increase in body height [29]. In this study, a decrease in acromiale height was observed in both groups during pregnancy, with its peak occurring at 28 g.w. During the postpartum period, increase in acromiale height of the experimental group may be associated with the flatfoot preventive effect of tested footwear, as the decrease in the foot arch during pregnancy has been reported previously [1, 2, 6] and a second-degree flatfoot has been associated with increased lumbar lordosis and thoracic kyphosis in young adult females [32].

### Hip joint

In the experimental group, statistically significant difference was found between 14 g.w. and postpartum. Postpartum, an increase in maximal hip flexion was observed. During pregnancy, an increase in hip flexion has been commonly reported previously and associated with the development of lower back, pelvis hip or sacroiliac pain [29, 33–35]. However, in the postpartum period in comparison with pregnancy, only a decrease in maximal hip flexion or no change in the hip range of motion was reported in previous studies [27, 29, 34]. In the experimental group, a statistically significant decrease in step cycle time was observed postpartum, suggesting an increase in gait velocity. Previous studies focused on the relationship of kinematic parameters and gait velocity demonstrated an increase in maximal hip flexion with increased gait speed [36, 37]. Therefore, the observed increase in hip flexion at the postpartum data collection session might be a reflection of the increased gait speed.

In the control group, the hip joint angle mean and maximum values were significantly increased at 28 g.w., suggesting an increased extension in the hip joint during this period of pregnancy. Similarly, increase in hip extension at the second trimester of pregnancy compared to the last trimester was found in the study by Branco et al. [26].

### Knee joint

During postpartum data collection session, in comparison with pregnancy, a decrease in maximal knee extension was observed in both groups. Additionally, an increase in maximal knee flexion at the advanced stages of pregnancy was observed in the control group. Similarly to this study, a greater maximum knee extension was observed in pregnant participants in a previous study comparing pregnant and non-pregnant women [38]. However, a decrease in maximum knee extension [39] as well as a lack of change in knee range of motion [27] were also observed in pregnant women previously. Similarly to the control group of current study, increase in the maximal flexion angle at advanced pregnancy was observed previously [39]. Increased knee flexion in the control group at the 37 g.w. might be connected to the foot arch decrease, observed during pregnancy in previous studies [1, 2, 6]. In kinetic chain studies, foot arch decrease has been associated with the increase in hindfoot pronation, observed in pregnant women [34], foot progression angle and calcaneus valgus. Hyperpronation in hindfoot induces increased shank internal rotation, compensatory knee flexion, femoral internal rotation and increase in anterior pelvic tilt and lumbar lordosis. Additionally, these changes may lead to pain development in the above mentioned body segments [2, 4, 32, 40–42].

Differences between the control and experimental group at the first data collection session in most of the analysed variables (anterio-posterior trunk angle, lateral trunk motion, spinal curvature change, knee joint angle, knee joint medio-lateral motion and knee joint height), as well as relatively high standard deviations of analysed variables indicate large individual differences in the gait pattern. Furthermore, the observed differences between right and left side, probably caused by the difference between the dominant and non-dominant limb, as well as differences between 28 and 37 g.w. might be the reason why the results of previous studies, including participants in different gestational weeks, are sometimes contradictory. Future studies focused on dominant and non-dominant limb differences will bring a more detailed insight into the pregnancy strategies during the gait.

There are some limitations of the study. All markers were placed on bony prominences; however, in a late pregnancy, those prominences were more difficult to palpate. A small sample size also constitutes one of the study limitations, as data variability of the experimental (n = 23) and control (n = 18) group indicate a highly individual gait patterns.

## Conclusions

The effect of tested footwear on kinematic gait pattern changes during pregnancy and postpartum period has been observed by the difference in gait pattern changes between the experimental and control group during pregnancy and postpartum period. These changes might be explained by the preventive effect of the tested footwear against the foot arches falling. Unlike the control group, in the experimental group, statistically significant spinal curvature straitening, increased maximal flexion in hip joint and decreased step cycle time were observed postpartum compared to pregnancy. In the control group, changes associated previously with the foot arches falling and hindfoot hyperpronation, such as increase in knee flexion or increase in spinal curvature, were observed during advanced phases of pregnancy and postpartum. However, differences between the control and experimental group at the first data collection session in most of the analysed variables, as well as relatively high standard deviations of analysed variables indicate large individual differences in the gait pattern, making the evaluation of tested footwear difficult. For the comprehensive evaluation of the tested footwear, future research combining the kinematic and dynamic plantographic methods is needed.

## Supporting information

S1 Table Results of statistical analysis for step cycle duration. (DOCX)
