# Supplementary material for: Special footwear designed for pregnant women and its effect on kinematic gait parameters during pregnancy and postpartum period
Source: PLoS One. 2020 May 12;15(5):e0232901. doi: 10.1371/journal.pone.0232901 (PMC7217473; doi:10.1371/journal.pone.0232901)
Supplement: S1 Table — (DOCX) [file pone.0232901.s001.docx]

S1 Table. Results of statistical analysis for step cycle duration.

|  | **Experimental group** | **Control group** |
| --- | --- | --- |
|  | **p value** | **p value** |
| **14 - 28 g.w.** | 0.922 | 0.093 |
| **14 - 37 g.w.** | 0.296 | 0.055 |
| **14 g.w. - postpartum** | 0.073 | 0.309 |
| **28 - 37 g.w.** | 0.372 | 0.795 |
| **28. g.w. - postpartum** | 0.006* | 0.740 |
| **37 g.w. - postpartum** | 0.004* | 0.653 |
